# Developing a data-driven spatial approach to assessment of neighbourhood influences on the spatial distribution of myocardial infarction

**DOI:** 10.1186/s12942-017-0094-8

**Published:** 2017-06-07

**Authors:** Wahida Kihal-Talantikite, Christiane Weber, Gaelle Pedrono, Claire Segala, Dominique Arveiler, Clive E. Sabel, Séverine Deguen, Denis Bard

**Affiliations:** 1 LIVE UMR 7362 CNRS (Laboratoire Image Ville Environnement), University of Strasbourg, Strasbourg 6700, Strasbourg, France; 2 UMR Tetis (Territoires, environnement, télédétection et information spatiale), Montpelier, France; 3 The French National Public Health agency, Saint-Maurice, France; 4 SEPIA-Santé, Baud, France; 5 Department of Epidemiology and Public Health, EA 3430, FMTS, Strasbourg University, Strasbourg, France; 6 School of Geographical Sciences, University of Bristol, Bristol, BS8 1SS UK; 7 Department of Environmental and Occupational Health, School of Public Health (EHESP), Sorbonne Paris Cité, Rennes, France; 8 Department of Social Epidemiology, Sorbonne Universités, UPMC Univ Paris 06, INSERM, Institut Pierre Louis d’Epidémiologie et de Santé Publique (UMRS 1136), Paris, France; 9 Department of Quantitative Methods in Public Health, School of Public Health (EHESP), Sorbonne Paris Cité, Rennes, Paris, France

**Keywords:** Data-driven, Multidimensional, Spatial approach, Neighbourhood influences, Social health inequalities, Myocardial infarction

## Abstract

**Background:**

There is a growing understanding of the role played by ‘neighbourhood’ in influencing health status. Various neighbourhood characteristics—such as socioeconomic environment, availability of amenities, and social cohesion, may be combined—and this could contribute to rising health inequalities. This study aims to combine a data-driven approach with clustering analysis techniques, to investigate neighbourhood characteristics that may explain the geographical distribution of the onset of myocardial infarction (MI) risk.

**Methods:**

All MI events in patients aged 35–74 years occurring in the Strasbourg metropolitan area (SMA), from January 1, 2000 to December 31, 2007 were obtained from the Bas-Rhin coronary heart disease register. All cases were geocoded to the census block for the residential address. Each areal unit, characterized by contextual neighbourhood profile, included socioeconomic environment, availability of amenities (including leisure centres, libraries and parks, and transport) and psychosocial environment as well as specific annual rates standardized (per 100,000 inhabitants). A spatial scan statistic implemented in SaTScan was then used to identify statistically significant spatial clusters of high and low risk of MI.

**Result:**

MI incidence was non-randomly spatially distributed, with a cluster of high risk of MI in the northern part of the SMA [relative risk (RR) = 1.70, p = 0.001] and a cluster of low risk of MI located in the first and second periphery of SMA (RR 0.04, p value  =  0.001). Our findings suggest that the location of low MI risk is characterized by a high socioeconomic level and a low level of access to various amenities; conversely, the location of high MI risk is characterized by a high level of socioeconomic deprivation—despite the fact that inhabitants have good access to the local recreational and leisure infrastructure.

**Conclusion:**

Our data-driven approach highlights how the different contextual dimensions were inter-combined in the SMA. Our spatial approach allowed us to identify the neighbourhood characteristics of inhabitants living within a cluster of high versus low MI risk. Therefore, spatial data-driven analyses of routinely-collected data georeferenced by various sources may serve to guide policymakers in defining and promoting targeted actions at fine spatial level.

## Background

Despite a succession of high-profile reports based on scientific studies demonstrating the links between social determinants and several health outcomes, health inequalities persist and still constitute a major public health issue [1–3]. Since the early 2000s, there has been a growing number of studies demonstrating the role played by ‘place’ where people live (also referred to as ‘context’) in influencing health status [4–7]. More precisely, the underlying idea is that the health effect of the environment exposure is complex, including both direct effect of specific environmental exposure (e.g. air pollution) and indirect consequences commonly addressed as the concept of “neighbourhood” [4, 6–8]. Many literature reviews support the significant effect of neighbourhood on a set of outcomes [9] such as mental health, birth [10], early childhood health [11], and obesity [12].

In order to explain the pathway via which neighbourhood may affect health, several papers have proposed conceptual models related to neighbourhood and to individuals’ behaviours—such as physical activities [13], walkability [13], diet [14] and such bio-physiological events as stress [15]. For instance, the causal framework proposed by Pearce et al. [16] uses three distinct domains to describe the various components of neighbourhood: physical characteristics (quality of outdoor environment and housing, traffic and physical disorder, etc.), (2) social characteristics (social network, social cohesion, etc.), and (3) community resources access (leisure facilities, healthcare, etc.). More recently, Komeily et al. [17] have defined neighbourhood as a function of several variables selected from physical (street design, connectivity, building type and use, etc.), operational (transit stops, routes, etc.), socioeconomic (demographics, land use and density, etc.) environmental (climate, topography, etc.) and institutional points of view (policy, etc.). In the majority of studies, however, neighbourhood was characterized by a single variable such as, for instance, noise [18–20] or the presence of graffiti, [21] defining the physical domain in epidemiological studies investigating respiratory [18] or cardiovascular disease [18–20]. Characterization of neighbourhood in the domain of community resources access, food store accessibility [22], primary healthcare services, recreational facilities, and public open [23, 24] and green spaces [25, 26] has been investigated in the literature. The role of the social domain has so far been explored mainly through data on local violence [27, 28] and social cohesion (or social capital) [29].

Each of these domains has been recognized as being associated with health status beyond socioeconomic status. For instance, the association between a low social standing measurement for residential neighbourhood and blood pressure was found after adjusting for individual/neighbourhood socioeconomic status and individual risk factors for hypertension [30]. A recent systematic review revealed that the majority of studies show a reduced risk of cardiovascular disease mortality in areas having higher residential greenness [31]; a finding confirmed by another study investigating respiratory disease, which showed that children living in areas with more street trees have lower prevalence of asthma [32]. In addition, certain neighbourhood characteristics–such as proximity and/or access to green space or healthcare–are often not equitably distributed with regard to socioeconomic status [33]—and this could exacerbate health inequalities.

Fine neighbourhood characterization for the study of health effects now has major policy implications for the public health community, to promote development and application of policies and social action aimed at reducing health inequalities [34–36]. Moreover, the spatial identification of small geographical areas carrying a high health risk, and their contextual characteristics, could allow for action more closely targeted at those most at risk [37, 38].

In this context, the issue is the definition of relevant, evidence-based public health interventions, armed with precise knowledge of what truly influences health inequalities in a given setting and among specific, vulnerable population groups. It should be stressed that such knowledge may inform the “Health in all Policies” strategy advocated by WHO and the European Union [39, 40], through actions on urban planning, transport, educational services, social work, and amenities (including leisure centres, libraries and parks).

In this work, we sought to combine a data-driven approach with clustering analysis techniques, to investigate neighbourhood characteristics (including socioeconomic and public resources as well as the psychosocial dimension) that may explain the geographical distribution of onset of MI risk. This work is not intended to reveal any relationship or causal pathway between neighbourhood characteristics and MI risk; other, more appropriate studies were designed to answer this question [9].

## Methods

### Study setting

Our study setting was the Strasbourg metropolitan area (SMA), an urban area of 316 km^2^, located in the Bas-Rhin district of the Great-East region of north-eastern France, and having a population of 500,000. This area comprises 33 municipalities subdivided into 190 French census blocks named IRIS (Ilots Regroupés pour l’Information Statistique), each having an average of 2000 inhabitants.

This French census block/IRIS (a sub-municipal French census block) is defined by the National Institute of Statistics and Economic Studies (INSEE). This is the smallest administrative unit in which socioeconomic and demographic data are available in France. In terms of population size, French census block is intermediate between US census tracts (about 4000 inhabitants) and US census block groups (about 1000 inhabitants).

### Neighbourhood characteristics

To our knowledge, few groups have attempted to combine all the domains addressed above [41, 42]. For instance, the UK Department of the Environment, Transportation, and the Regions (DETR) [42] developed an Index of Multiple Deprivation (IMD) as an official measure of relative deprivation for small areas (or neighbourhoods) in England—based on a combination of six or seven domains.

As in the British contextual frameworks, we have undertaken a process of characterizing a neighbourhood in the SMA that includes the most common domains capable of supporting health studies of related to: socioeconomic, community resources (or public resource), and psychosocial (or social).

### Data sources: Table 1

All socioeconomic data including employment, educational level, income, data about those receiving child benefit and also those receiving the French welfare allowance was obtained from the French National Census Bureau (INSEE-*Institut National de la Statistique et des Etudes Economiques*) and from the statistics department of the CAF (Caisse d’Allocations Familiales), family welfare system.

**Table 1:** Data source to characterize the neighbourhood context

Domain	Category	Variables	Provider (Year)	Exhaustivity of location data
Domain 1: socioeconomic environment	Population	Total population % of born abroad	French National Census Bureau (INSEE- Institut National de la Statistique et des Etudes Economiques) (1999)	
	Employment	Unemployment rate % of Blue collars among the active population with permanent jobs Non-permanent job rate		Data available from census block level
	Education	% Persons aged 15+ without qualification		–
		People aged 15 years or older with at least a lower tertiary education		
		People aged 15 years or older who did not go beyond an elementary education		
	Family	% of single-parent families		
	Household	% of households with no car % of households with 2 cars		
	Income	% of population entitled to family allowance	Statistics department of CAF (Caisse d’Allocations Familiales) (2007)	
		% of population entitled to safety net income		
Domain 2: public resources	Healthcare system	Location of doctors’ surgeries—Location of healthcare centres	Regional health agency/French National Directory of Health and Social Establishments (2007)	Systematic census of all doctor and healthcare centre addresses located in the SMA
	Public transportation supply	Location of bus and tram stop and the number of lines served at each	SMA authority (2008)	Exact location ground-truthing
	Public parks and gardens	Location and area of public parks and gardens	SMA authority et CIGAL Spatial Data Infrastructure (Coopération pour l’Information Géographique en Alsace) (2008)	Systematic census conducted by the SMA authority (using ground-truthing) of all public parks (where inhabitants may practice sport)
	Sport facilities	Location of sport facilities	Great-East regional and district office DRDJS (Office of Youth and Sports) (2008)	Systematic census of all sports facilities by the Office of Youth and Sports, using ground-truthing
Domain 3: psychosocial environment	Local businesses	Location of retail outlets Location of food markets	SMA authority (2008)	Systematic census of retail outlets and food markets conducted by the SMA authority using ground-truthing of itinerant vendors only (small markets)
	Characterization of educational facilities	Number and type of Violence in schools	Official education institutions (Ministère de l’éducation). (2007)	
		Schools’ social scores	Inspection d’académie (Ministère de l’éducation) (2007)	Exact location and characteristics of education facilities provided by the official educational institutions that manage these schools
		Primary/middle and secondary (high) schools ZEP (priority) and successful (AR) middle schools	SMA authority and official education institutions (2007)	
		Map showing primary and middle schools Secondary (High) schools	General Council of the Bas-Rhin and official education institutions (2007)	
			SMA authority and official education institutions (2007)	
	Voting rates	Voting rates	The City Halls of Strasbourg (2000–2008)	–
	Civic associations	Location of civic associations	SMA authority and SIRENE databases (2000–2008)	
		Type of civic associations: Religious, political, volunteer		Exact location of association without use of ground-truthing

*SMA* Strasbourg metropolitan area

To characterize access to public resources, the regional health agency provided all the FINESS (French National Directory of Health and Social Establishments) files, which describe the healthcare system (physicians and facilities). The SMA made geocoded data available that allowed us to determine (1) transportation elements such as bus and tram stops and the number of lines served, as well as (2) geocoded data on location of public parks and green spaces. Lastly, the Great-East regional and district office DRDJS (Office of Youth and Sports) made available its database of all athletic equipment and facilities. However, no information concerning the usage of amenities was collected in this study.

To characterize the psychosocial environment, including the civic and community environments, local businesses and retail stores, and educational environment, we used SIRENE databases (INSEE), the educational facilities database available at the SMA authority and official education institutions, as well as data provided by the city’s list of itinerant vendors (small markets). The CIGAL Spatial Data Infrastructure (Cooperation pour l’Information Géographique en Alsace), provides a database describing land use and land cover coverage and categories (see Table 1).

### Geographical information system analysis

Of the databases collected, some datasets were available at administrative spatial base level—such as census block. Such segmentation might, however, not be relevant for spatial analysis of other data produced for different purposes, at various scales. Instead of using the available French census block files, we therefore chose to design a specific spatial unit mesh, allowing us to manage the data’s scale heterogeneity (that is, a square grid) for three reasons:

- Stability of the basic geographical unit; one advantage of cell-based over administrative borders (likely to change over time) is that it can be fixed: its borders do not change over time unless desired—in response for example to changing underlying population or land-use footprints.
- Administrative spatial units and their borders are not necessarily relevant for subsequent analysis other than that for which they were constructed.
- To homogenize contextual data; contextual data is extremely heterogeneous in terms of spatial scales, collection dates, and exhaustiveness. Use of the grid makes it possible to homogenize data to some extent, ahead of any statistical or spatial analysis.

To determine grid path size, we used the “nearest neighbour” method [43] to characterize the spatial distribution of the different patterns of geographical points (retail store, physicians, etc.). The mean distance separating points has been calculated as 270 m. Cell dimension was thus set at 250 m × 250 m to best approximate underlying data distribution, yielding 5127 cells for the SMA coverage. All contextual variables collected were assigned at this cell level.

Zonal data (such as the socioeconomic data obtained at IRIS scale for the 1999 census) was fitted to the 250 × 250 m grid using a clipping function. The “zone clipping” algorithm is then used to disaggregate the variable, according to a geometric overlap principle. The value of the information transferred to the cell is thus a function of the area common to the initial area (for example, the IRIS) and the grid cell.

In this desegregation approach, we assume equal density of the phenomenon across the area. The space considered, however, is not isotropic. This constraint was overcome using available geographic information (topographic database) to improve characterization of the disaggregation of the initial area.

In our study, we postulate that the equidistribution of data was a function of the buildings’ volume: in this case, we estimated the population of the cells proportionally to the habitable area of the buildings included in the cells, according to the following formula:

$$ {\text{Population}} = \sum\limits_{\text{N}}^{{{\text{i}} - 1}} {{\text{P}}_{\text{IRIS}} }_{\text{i}} \times \frac{{\sum\nolimits_{\text{n}}^{{{\text{i}} - 1}} {{\text{Area}}\;{\text{of}}\;{\text{housing}}_{\text{i}} } }}{{{\text{Total}}\;{\text{area}}\;{\text{of}}\;{\text{housing}}\;{\text{in}}\;{\text{IRS}}_{\text{i}} }} $$

where Area of housing = Building footprint area of housing × Number of habitable floors. Number of habitable floors = housing height/3.

Once all socioeconomic variables had been desegregated at cell level, we calculated the socioeconomic indicator for each cell (e.g. unemployment rate, % of blue-collars among the active population with permanent jobs, non-permanent job rate).

For all spatial analyses described below, each cell was represented by the centroid of the inhabited built area.

### A data-driven approach to neighbourhood characterization

First, the 25 variables described in Table 1 were geolocated and analysed in line with the approaches proposed by various studies (Table 2).

**Table 2:** Spatial characterization of different field of neighbourhood

Domain	Category	Variables	Spatial shape	Geographic Information System (GIS) analysis
Domain 1: socio-economic environment	Population	Total population All socio-economic variables	Zonal data available at census block level (2000 inhabitants on average)	Using the ArcGIS software zone-clipping algorithm, we disaggregated the variables according to real weighting interpolation methods. Because the value of the information transferred to the cell was thus a function of the area common to both the initial area (here, the census block) and the grid cell, these variables were able to be integrated into the final analysis
Domain 2 : public resources	Healthcare system	Location of doctors’ surgeries Location of healthcare centres	Point data: address	We assigned to each cell centroid the road distance (non-Euclidian) to the nearest healthcare centre or doctor’s surgery
	Public parks and gardens	Location and area of public parks and gardens	Polygon data: Location and size	We built an attractiveness index for public parks and gardens, derived from French studies showing that attractiveness is a function of size. Using GIS tools, we drew concentric zones of attractiveness by area: 100 m (area less than 1 ha), 500 m (area 1–10 ha), and 1000 m for larger areas. We subsequently computed this index for each cell
	Sports facilities	Location of sport facilities	Point data: address and coordinate X, Y	The road network distance to the nearest sports facility was attributed to each cell centroid
	Public transportation supply	Location of bus and tram stop and the number of lines served at each	Point data: coordinate X, Y	Using GIS tools, and on the basis of modal differential attractiveness between these two types of public transportation, we constructed a public transportation availability indicator, with a catchment area attributed to each stop (300 m for a bus stop, 400 m for a tram station), weighted by the number of lines at each stop or station. This indicator was then assigned to each cell
Domain 3: psychosocial environment	Local businesses	Location of retail outlets	Point data: address and coordinate X, Y	Using GIS tools, we attributed to each unit the quantity of retail stores relative to all available retail space within a radius of 200 m around the spatial unit centroids. The resulting values associated with the retail store scoring (quantity of retail stores relative to all available retail space) by category (itinerant vendors; retail food stores; retail non-food stores and other services) were attributed to each unit^a^
		Location of food markets	Point data: address and coordinate X, Y	
	Characterization of educational facilities	Violence in schools Schools’ social scores Primary/middle and secondary (high) schools ZEP (priority) and successful (AR) middle schools Map showing primary and middle schools Secondary (High) schools	Point data: address and coordinate X, Y	The French school environment is graded as: (1) Priority education zones (ZEP-*Zone d’éducation prioritaire*), where establishments receive additional resources and have greater autonomy for dealing with educational and social difficulties, (2) “successful ambition” zones (AR), having fewer (but definite) needs and thus fewer resources), and (3) others. All non-private schools in the city and their catchment area were geocoded, using information provided by local authorities. We computed an indicator taking into account school density and classification (primary/middle or secondary/high schools)
	Voting rates	Voting rates	Zonal data available for each center of vote	
	Civic associations	Civic associations	Point data: address and coordinate X, Y	The fairly exhaustive and georeferenced SIRENE database allowed calculation of the ratio of the number of (official) civic associations per 100 inhabitants in each unit, taking into consideration their type (religious, political, other)
		Type of civic associations: religious, political, volunteer	Point data: address and coordinate X, Y	

^a^200 m is the distance for which 50% of the cells have at least one market

Second, we aimed to create a multidimensional profile with which to characterize each neighbourhood based on the underlying data structure using a data-driven approach, and without any a priori models.

Consider a data set composed of each domain within the same unit as group of variables. As we had several groups of both quantitative and qualitative contextual variables (socioeconomic, public resource, psychosocial) and because we wanted to give each equal weight regardless of the number of variables in it, we used Multiple Factor Analysis (MFA) [44]—a technique well-suited to this situation.

The MFA entailed performing either a Principal Component Analysis (PCA) for each subset, if the group is composed of quantitative variables (sets of both socioeconomic and public resources domain variables), or a Multiple Correspondence Analysis (MCA) if the group is composed of qualitative variables (sets psychosocial domain of variables). This first step allowed us to compute distance between units by giving a specific weight to each variable, based on use of the highest eigenvalue of the PCA or the MCA for each group, thus obtaining a particular metric. In the second step of the MFA, we used the previously obtained metric to perform a PCA on the whole data set. This allowed us to compare groups of different types of variables.

Following the MFA, we applied Hierarchical Ascendant Clustering (HAC) [45] to create meaningful contextual profile (cf. Appendix for Fig. 4). HAC is an unsupervised clustering method that creates a hierarchy of classes (clusters), and is frequently used after MFA. Given a set of variables created by the MFA, the HC algorithm creates a hierarchy of categories, step by step—at each step merging the two categories that are closest, according to a given distance between categories. When it is a particular distance (Ward distance), this algorithm aims to obtain categories that are homogeneous within and heterogeneous between one another, with respect to an inertia-based criterion.

These approaches therefore allow us to build a partition of our unit into homogeneous clusters (low within-variability) that are different from one another (high between-variability), ultimately producing a categorical indicator, referred to in our previous work as the Neighbourhood Deprivation Index (NDI) [46] (for more detail, see Sabel et al. [46]). These analyses were performed using SPAD 7.0 statistical software.

### Synthetic neighbourhood design

To evaluate spatial implication of neighbourhood planning, we have chosen to define specific boundaries of the neighbourhood, so as to use (1) a more homogeneous area (with high intra-zone homogeneity and inter-zone heterogeneity), and (2) an area with population size set to 2000 inhabitants, similar to the French census blocks, ensuring health data confidentiality.

To produce these synthetic neighbourhoods, we used the AZTool zone design program provided by David Martin (University of Southampton, UK) to aggregate contiguous and homogeneous spatial units (cells) for generating optimal geographies [47, 48]. To produce a synthetic homogeneous neighbourhood, three criteria were considered: (1) output zone homogeneity (and inter-zone heterogeneity), using our NDindex as the homogeneity criterion; (2) population target size equal to 2000 inhabitants (similar to French census blocks) to ensure health data confidentiality; (3) shape compactness, avoiding linear or quasi-linear output zones. To design the new zones, we used different combinations of relative weighting of parameters (criteria) in the AZTool (population target, shape and homogeneity) to create candidate sets of pseudo-blocks (in total six experimental conditions were tested). To improve AZT performance, we used simulated annealing (SA). Next, we evaluated the zonal system (each criterion defined below) to identify the optimal solution using a measure of within-area homogeneity (IAC) and measure shape compactness (P2A score) for each experimental condition. International experience and AZTool parameter setting advice accepts an IAC of greater than 0.5 as representing a very reasonable degree of homogeneity. Then, to improve AZT’s solution and the found optimum solution, we sought to optimise two conditions for which IC >0.5 and which also presented a shape that was more compact than linear, by increasing the number of iterations. For more details, see Sabel et al. [46].

### Health data: MI

All MI events [International Classification of Diseases, 9th Revision (ICD-9): 410] occurring in the SMA, among the population aged 35–74 years, collected by the Bas-Rhin coronary heart disease register [49] between January 1, 2000 and December 31, 2007 were geocoded at their residential address areal unit (see below). Specific annual rates, standardized by age and gender (per 100,000 inhabitants), were calculated for each neighbourhood by contextual profile. Khi^2^ tests were performed to compare the annual rate between the five contextual profiles.

## Spatial method

In order to explore the geographic pattern of the MI risk, we used the spatial scan statistics (implemented in the SaTScan software [50]) to statistically and significantly detect the presence of potential clusters for both high and low risk. This approach, used in an increasing number of applications in the field of spatial epidemiology [51–55], allowed us to (1) identify the specific spatial location of the clusters and (2) evaluate and understand the implications of neighbourhood characteristics in the spatial distribution of MI risk [56, 57].

The procedure works as follows: a circle (or windows) of variable radius (from zero up to 50% of population size [56]) is placed at every centroid of the synthetic neighbourhood and moves across the whole study area to compare the MI rate in the windows with what would be expected under a random distribution.

In our study, the Poisson probability model implemented in the SaTScan software [50] was chosen as *cluster analysis method*. The number of cases in each census block is assumed to follow a Poisson distribution. Our cluster detection approach identified clusters of both high and low rates with maximum circle window size, to include up to 50% of the population at risk. Identification of the most-likely clusters is based on a likelihood ratio test [56] with an associated p value obtained using Monte Carlo replications [58]. The number of Monte Carlo replications was set to 999 to ensure adequate power for defining clusters and considered a 0.05 level of significance (p value derived from 999 replications).

If we detect a significant most-likely cluster (with p < 0.05) using this method, a logical next step is to take account of the individual characteristics acknowledged in the literature and available in our studies, to see whether the significant cluster can be explained by suspected risk factors. Spatial analyses were thus performed in two stages (step by step):

1. Unadjusted analysis, to identify and localize the most-likely cluster of high/low risk of MI.
2. Analysis adjusted for age and sex included this information directly in the SaTScan model [50].

## Results

The MFA was applied on the 27 selected variables covering the three contextual groups described above. The first four components explain only 17, 8, 5 and 5% of total variance respectively (Table 3). These components can be interpreted using the contributions made by both groups and variables to the components or their graphical representations. To explain 60% of total variance, we needed to use ten components, because all ten were used as a basis for the HC in order to preserve all the variability of the initial information.

**Table 3:**
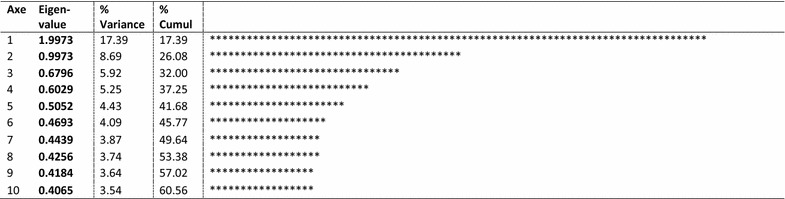
Eigenvalue and variance explained by the ten first components of the MFA

**Fig. 1:**
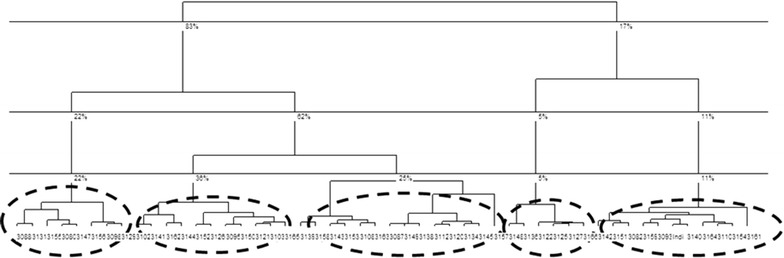
Dendrogram showing the classification of 5 contextual profiles

In line with the MFA, we performed an HAC—and according to both the dendrogram and the Ward distance (Fig. 1), we chose a 5-category partition. From the HAC analysis, then, five clusters (or contextual profiles Table 4), were determined using the coordinates of the cells for the first ten factorial axes of the MFA. Using the characteristics of each category by variable (Table 4), five contextual profiles can be identified in the SMA.

**Table 4:** Description of neighbourhood characteristics of five contextual profiles

	Class A	Class B	Class C	Class D	Class E
*Socio economic feature*					
Proportion of population covered by CAF	42.2%	44.7%	50.24%	51.40%	62.16%
Proportion of population covered by RMI	1.9%	1.5%	5.19%	4.64%	10.88%
Population density	71.13	180.24	556.10	706.04	470.48
Proportion of precarious jobs	8.62%	9.33%	13.32%	14.46%	16.58%
Proportion of stable jobs	76%	75%	68%	65%	59%
Unemployment rate	5.95%	6.61%	10.04%	11%	19.83%
Proportion of blue-collar workers	18.77%	18%	17%	16%	32%
Proportion of high school graduates	10.38%	10%	6.68%	9.70%	5.29%
Proportion of single-parent families	8.19%	9.11%	13.01%	13.5%	19.79%
Proportion of foreigners	4.03%	4.5%	8.79%	9.45%	17.60%
Proportion of people without cars	9.02%	10.5%	23.38%	30.6%	29.04%
Proportion of people with 2 cars	43.54%	38.41%	20.69%	17.05%	17.64%
*Access to resources*					
Availability of green space	5.48	2.06	4.75	8.89	6.91
Distance to healthcare facilities (m)	−1385.55	478.25	263.71	214.88	399.00
Public transportation coverage	2.28	7.75	20.88	23.19	15.12
Distance to sports facilities (m)	996.96	522.37	353.44	339.95	349.59
*Psychosocial environment*					
Quantity of civic associations	Very low	Low	high	Very high	Medium
Local school socio-educational classification	Very high	High	Low	Medium	Very low
Local retail store score	Very low	Low	High	Very high	Medium
Urban fabric (housing types)	Single-family homes	Mixed buildings	Mixed buildings	Center-city homes and Mixed	Multiple-dwelling unit buildings

Very high: very good social support, high: good social support; low: low social support; very low: very low social support

The first two axes of the MFA explained 29.14% of the variance. From the HAC analysis, 5 clusters or contextual profiles were determined from the coordinates of the cells for the first ten factorial axes of the MFA, so as to preserve all the variability of the initial information

*CAF* fund for family allocations, *RMI* minimum insertion income

In total, we have identified: Two profiles (A and B) characterized by favourable socioeconomic conditions, low psychosocial cohesion, and poor access to public resources; two profiles (D and E) characterized by low socioeconomic conditions, very strong psychosocial cohesion and very good access to public resources, and profile (C) characterized by medium socioeconomic conditions, high psychosocial cohesion and average access to public resources.

Table 4 shows neighbourhood characteristics for the five contextual profiles, determined through multidimensional analysis (MFA and HAC).

**Fig. 2:**
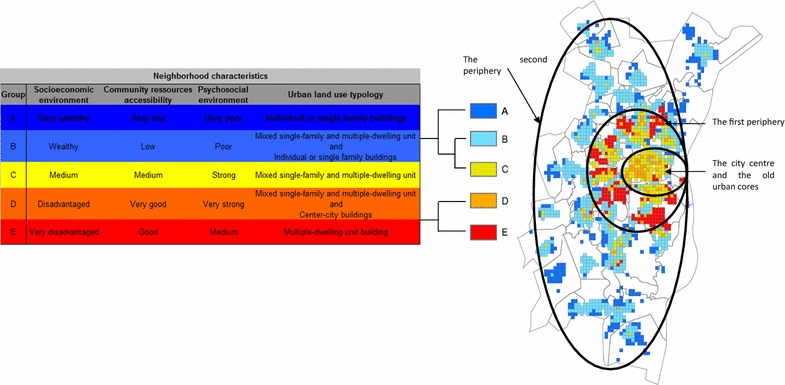
Mapping of the deprivation profile of the 5 categories of neighborhoods identified by the Hierarchical Ascendant Clustering (HAC)

Figure 2 shows the spatial distribution of these five contextual profiles from ‘A’ (least deprived) to ‘E’ (most deprived). Mapping these profiles shows that neighbourhood planning is spread unevenly across our study area. We have highlighted a centre-periphery gradient with two groups (C and D) characterizing the city centre and the old urban cores. A first periphery of SMA (profile E) concentrated on inner city neighbourhoods, which tend to be more distant from the historic city centre. A second periphery of SMA (profiles A and B) correspond to the urban extensions of the last decade and the urban spread in the SMA.

Table 5 presents the age-standardized mean annual rates (per 100,000 inhabitants) by gender and by neighbourhood contextual profile. Regardless of contextual profile, MI rates in women are always lower than those in men, at all ages, and MI rates are always much higher among the elderly. Secondly, profile A and B neighbourhoods are characterized by lower rates than the other profiles. Finally, MI rates differ significantly between contextual profiles among women.

**Table 5:** Distribution of myocardial infarction event rates according to contextual profiles

Mean annual event rates, per 100,000 (CI 95%)	A	B	C	D	E	p values^*^
*Neighbourhood contextual profiles (years)*						
Females 35–74	382 (240–523)	383 (333–466)	459 (381–537)	548 (402–694)	720 (600–840)	0.0008**
35–54	88 (2–174)	143 (98–201)	204 (137–271)	175 (72–278)	430 (314–546)	0.0121**
55–74	859 (515–1202)	777 (654–961)	855 (685–1025)	1202 (843–1562)	1241 (977–1505)	0.0320**
Males 35–74	1424 (1147–1702)	1612 (1540–1822)	1773 (1610–1936)	1678 (1411–1944)	2171 (1955–2387)	0.0794
35–54	737 (486–989)	834 (743–997)	1230 (1062–1398)	1112 (849–1374)	1283 (1079–1488)	0.2081
55–74	2601 (1983–3219)	2980 (2787–3423)	2785 (2440–331)	2909 (2283–3535)	3880 (3386–4374)	0.2104

* Khi^2^ test

** Significant p value <5%

### Identification of MI risk cluster

Spatial distribution of MI risk is not random, either across all SMA or between the five contextual profiles.

**Fig. 3:**
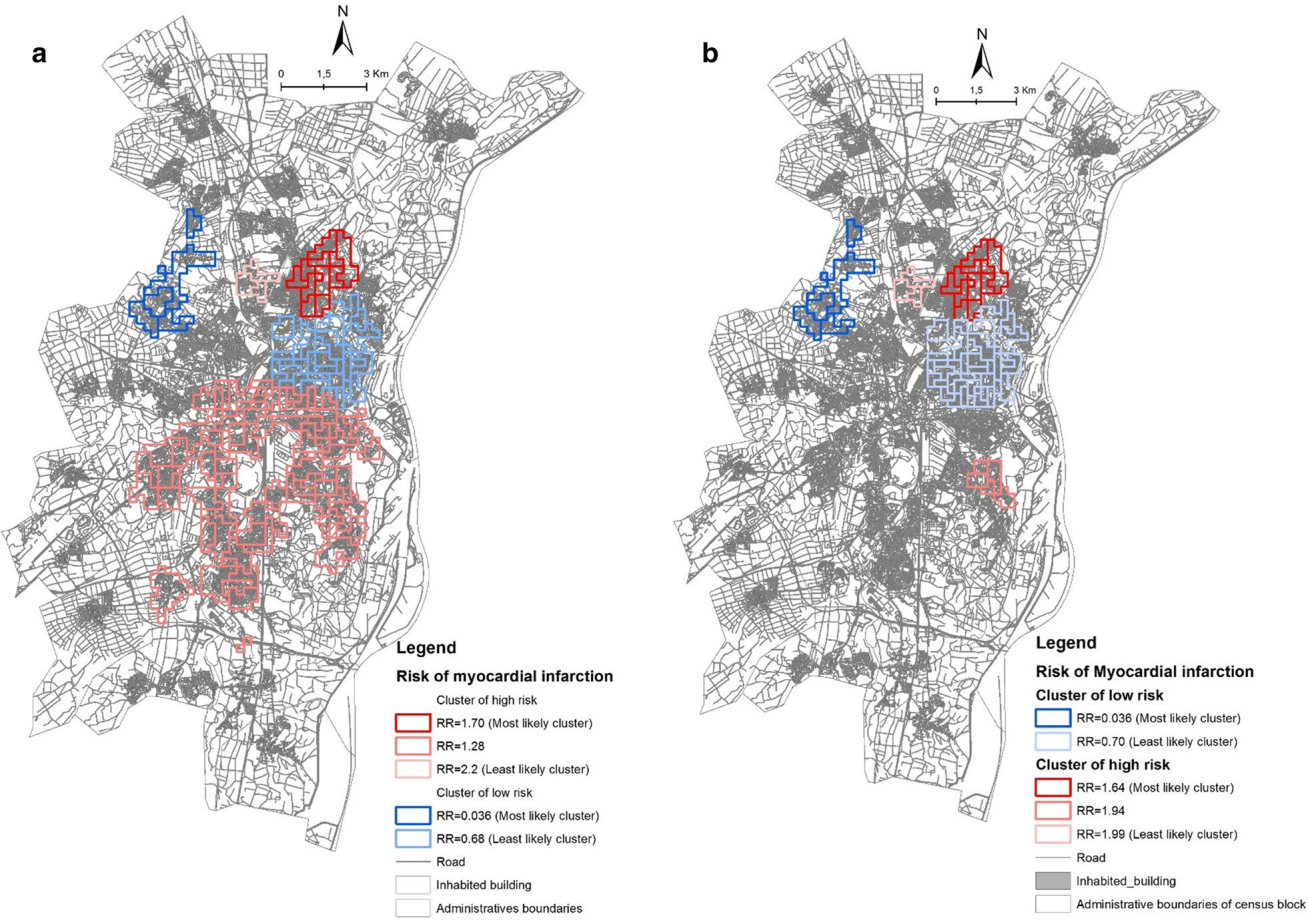
Spatial location of significant Clusters of high risk of myocardial infraction (in *red*) and low risk of myocardial infarction (in *blue*) identified in Strasbourg metropolitan area **a** crude analysis; **b** adjusted analysis on age and sex

**Fig. 4:**
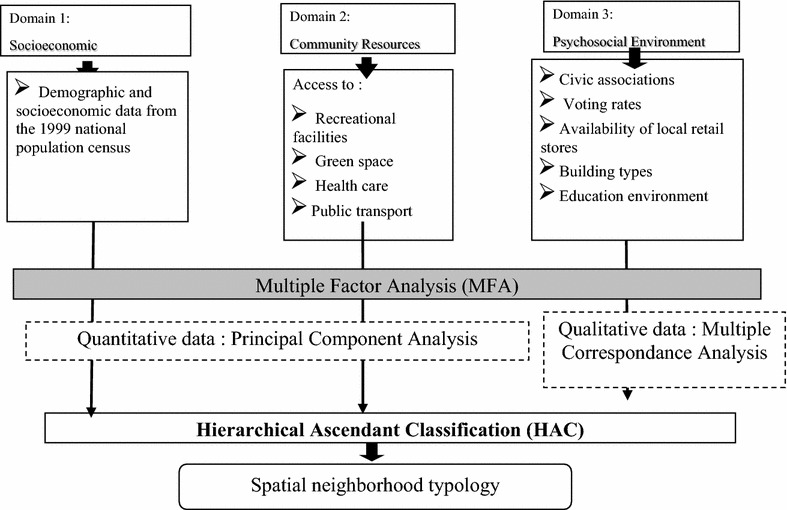
The construction of homogeneous neighbourhood categories

We identified three spatial clusters of high risk of MI (Fig. 3; Table 6) located mainly in the Strasbourg centre and first periphery of Strasbourg. These clusters are presented in order of most-likely cluster to least likely cluster in Fig. 3. Risk in the most-likely cluster (in the northern SMA) is 1.70 times greater than in the rest of the study area (p value  =  0.001). The second cluster, also identified within the northern part of the metropolitan area (RR  =  1.28) was not statistically significant, while the third cluster was located in the southern part of the metropolitan area (RR 2.02). After adjustment for gender and age group, we found the same most-likely cluster [relative risk (RR) 1.64; p value = 0.001] with a slightly lower likelihood value (down from 22.56 to 19.73), indicating that age and sex can explain some of the excess risk of MI observed in the unadjusted analysis (Fig. 3).

**Table 6:** The most likely clusters of high and low risk

	Radius (m)	Area included/population	Expected cases	Observed cases	RR^a^	LLr^b^	p value
Most likely cluster of high risk	1207.74	10/11,486	125.68	205	1.70	22.56	0.001
Most likely cluster of low risk	1978.61	5/5018	54.91	2	0.036	46.95	0.001

^a^ *rr* Relative risk

^b^ *LLr* Log likelihood ratio

On the other hand, we identified two spatial clusters of low MI risk (Fig. 3; Table 6) located mainly in the Strasbourg first and second peripheries. These clusters are presented from most-likely cluster to least likely cluster in Fig. 3. The most-likely cluster, in the western SMA, has lower risk that than in the rest of the study area (RR 0.04; p value  =  0.001). The second cluster was also in the northern part of the metropolitan area, and was also statistically significant (RR 0.68; p value  =  0.001). After adjustment for gender and age group, we found the same most-likely cluster, with a slightly lower likelihood value decreasing from 46.94 to 46.19 (Fig. 3).

### Spatial implication of neighbourhood characteristics of the clusters

In the clusters for high MI risk, the population profile is mainly ‘D & E’ which is socioeconomically very disadvantaged, with weak psychosocial cohesion and good access to public resources (see Tables 2, 7). Thus, compared to inhabitants in the rest of the study area, people living in those clusters identified as high MI risk, which had the highest proportion of population covered by welfare benefits (family allowances/child benefits, and the French “safety net” welfare allowance for people with resources below the poverty line), high rates of insecure employment, and the highest proportion of foreigners. These spatial units are characterized by good access to sports facilities and high retail store scores. This group is distinguished by the highest availability of green spaces, high public transportation coverage and weak community/civic fabric.

**Table 7:** Comparison between neighbourhood characteristics of inhabitant of cluster of high risk and inhabitant of cluster of high risk

Main characteristics	Most likely cluster		p value*
	Cluster of high risk^a^	Cluster of low risk^b^	
No civic associations	1.2%	99%	<0.0001
No school graded ZEP^c^	22.11	96%	<0.0001
Proportion of population covered by CAF higher that 60%	67%	13.62	<0.0001
Multiple–dwelling unit buildings	58.79	2.90	<0.0001
Single–family homes	24.6	90.43	<0.0001
Distance to healthcare facilities (<500)	76.8	4.93	<0.0001
No public transportation	10	60	<0.0001
Availability of green space	26	14	<0.05

^a^Neighbourhood characteristics of profile “E” and “C” which composed cluster of high risk

^b^Neighbourhood characteristic of profile “A” which composed cluster of low risk

^c^ *ZEP* Priority education zones: where establishments receive additional resources, and have greater autonomy for dealing with educational and social difficulties

* Khi test

However, in the low MI risk cluster, the population profile is mainly ‘A’—which describes the most socioeconomically advantaged areas having low psychosocial cohesion and very poor access to public resources (see Tables 2, 7). This most-likely cluster identified for low MI risk (n = 5018 inhabitants in the significant spatial clusters) had a significantly lower proportion of inhabitant rates of unemployment and of insecure (or temporary) jobs: on the contrary, the employment rate is stable and the proportion of high school graduates is highest. This group is characterized by the longest distances to healthcare facilities, and very poor access to public transport. It has an extremely favourable socioeconomic profile with low psychosocial cohesion and very poor access to public resources.

## Discussion

Our study confirms work we previously conducted on the SMA [59], which demonstrated that, whatever the level of deprivation, the rates of events in men were always clearly higher than those in women, at all ages. The literature reported that the relationship between neighbourhood characteristics may vary by gender, as our findings suggest. For instance, several studies have found stronger associations of neighbourhood characteristics with CHD outcomes in women than in men [60–62]. These gender differences could result from gender differences in health-related behavioural responses to neighbourhood perceptions. In addition, we observed a clear increase to the event rate with age, even after stratification by gender and deprivation.

Our study’s data-driven approach has allowed us to provide a fine description of the neighbourhood, using a set of contextual data. It highlights several neighbourhood profiles and provides us with evidence on the different combinations of dimensions within the SMA. In comparison with the literature, our profiles reveal differences—especially with regard to how the socioeconomic, social cohesion and access to amenities dimensions are combined.

Several studies show that individuals living in deprived socioeconomic environments have less access to businesses, sports leisure and other infrastructure. For instance, some have revealed that people living in deprived neighbourhoods are less likely to make use of green spaces because they do not perceive the need to do so [63, 64]. We revealed an inverse relation in the SMA: neighbourhoods with a deprived socioeconomic environment are characterized by a substantial presence of sports leisure infrastructure, unlike neighbourhoods with an advantaged socioeconomic environment.

Another aspect highlighted by the literature concerns the relationship between social capital and socioeconomic deprivation. Research projects have demonstrated that socioeconomic deprivation is associated with reduced levels of social capital [65]. Our study, however, shows the opposite result. In the SMA, neighbourhoods with an advantaged socioeconomic environment are characterized by a low level of social cohesion in comparison with neighbourhoods with a deprived socioeconomic environment, which are characterized by a high level of social cohesion.

Regarding the geospatial analysis performed (based on the Kulldorff approach), our study characterized the neighbourhoods of inhabitants living within a cluster of high MI risk, in comparison with those living within a cluster of low MI risk. Although our study allows us to precisely characterize the neighbourhoods included in the cluster with higher MI risk, it was not designed to reveal the MI risk factor among neighbourhood characteristics. Our spatial analysis is more suited to the formulation of certain hypotheses aimed at improving our understanding of the unequal spatial distribution of MI risk using the contextual data panel.

- First, the neighbourhood characteristics of inhabitants living within a cluster of high or low MI risk seem to have more disadvantaged and advantaged conditions respectively, confirming the results of previous studies [66]. Indeed, MI risk was significantly higher among: those whose education ceased after primary or secondary school, compared with those with a higher level of education (university) [66]; the unemployed [67], and men in the lowest socioeconomic group [68].
- Secondly, using only the accessibility and attractiveness of amenities indicator, our study revealed that within high MI risk clusters, inhabitants have excellent access to various amenities (including transport, green space and park and sports facilities)—in contrast to the low MI risk clusters. In the literature, results are contrasted depending on the measure used to describe availability/proximity of the infrastructure. For instance, some studies reported protective associations of green space against high blood pressure [69], coronary heart disease *and* cardiovascular disease mortality [70]. In New Zealand, however, Richardson et al. found no evidence that cardiovascular disease mortality was related to availability of either total or usable green space. In Tamosiunas et al. [71] found that the prevalence of cardiovascular risk factors was not related to the distance from people’s homes to green spaces—but was significantly lower among park users than among non-park-users.
- Lastly, the characterization of neighbourhoods of inhabitants living within a cluster of high MI risk show that they have high psychosocial cohesion in comparison with inhabitants within a cluster of low MI risk. This finding is incoherent with other studies which found that lower neighbourhood cohesion predicted higher coronary artery calcification prevalence [72].

## What this research adds in public health?

Beyond the geospatial approach applied on the local territory in France, this study answers to a major problem identified today by WHO to which classical epidemiological approaches do not respond. The European Union, supported by the World Health Organization (WHO), recognizes that it is time to move from the research about risk factors of health disparities to actions which aim to reduce them. Research conducted in public health policy issues supply little evidence for effective interventions aiming to improve population health and to reduce health inequalities.

This paper is attempts to fill the gap regarding a need for powerful tool to support priority setting and guide policy makers in their choice of health interventions, and that maximizes social welfare.

Today, more and more international and European institutions suggest certain actions on place that could improve health and thus tend to reduce health inequalities, such as improving access to, and quality of, green space, particularly in deprived areas—providing places for play, physical activity and favouring social interaction. For instance, the World Health Organization has also announced that access to green spaces can reduce health inequalities, improve well-being [73]. More recently, NHS Health Scotland stated, in the “Place and Communities Report” that policy and practice should continue to integrate health, housing, environment, transport, and community and spatial planning to improve health outcomes and promote sustainability [74].

In the majority of epidemiological research projects investigating health inequalities, sophisticated analyses are implemented to measure the strength of the association between risk factors and outcomes. These research findings may be pivotal to public health policy, but an attempt to distinguish between correlational and causal associations does not form the basis of effective interventions aimed at improving population health and reducing health inequalities. These classic epidemiological approaches offer limited guidance to policymakers in their choice of intervention, and suggest the need for spatial approaches to the investigation of social health inequalities.

Our study describes an approach that may guide policymakers in selecting which priority setting to use, and in choosing and developing the most appropriate local intervention if, for instance, they decide to apply the ‘proportionate universalism’ strategy described by Marmot in 2010. Policymakers are thus enabled to plan targeted interventions, choosing one of two appropriate broad approaches to action that are commonly accepted today as reducing health inequalities [36].

The present paper permits to novel way to investigate the social health inequalities:

1. Our work highlights that the investigation of the spatial distribution of multiple risk factors, including social, economic and contextual factors, can help policy makers to choose appropriately between two or more broad approaches which will be performed for the whole population, but with a scale and intensity proportionate to need.
2. The local diagnosis can assist policy makers to focus the scope of prevention/intervention programs and changes to the health care system, thus providing more effective interventions in order to response to individual needs, and public resources can be distributed more efficiently. Thereby, this spatial tool may assist the policy maker to tackle the social gradient in health if they choose to apply the strategy named ‘proportionate universalism’ and described by Marmot in 2010 [75].
3. In addition, our study show that the use of a routinely-collected data set within a data-driven approach to characterize neighbourhood, alongside a geospatial tool combined with GIS will be particularly relevant and of interest to policymakers involved in the identification, definition and promotion of targeted health inequality actions at varying spatial levels.
4. This study illustrates the usefulness of the geospatial approach using routinely-collected data to support policy makers in planning more focused community interventions in appropriate areas and to choose if public health interventions should be declined either at a national level, at a local level, or both.

### Strengths

The areal unit we constructed at a very small scale allowed us to consistently accommodate data produced at different scales. Our use of a single grid allowed us to minimize the effect of scale associated with the modifiable areal unit problem (MAUP), [76] because all the basic spatial units (cells) were constructed to have the same area. These new spatial units offer three benefits: (1) they make it possible to homogenize the best of the data collected, prior to any statistical or spatial analysis; (2) they allow us to spread the value of a piece of geographic information initially noted or represented according to a specific unit, in values calculated according to regular spatial units, while preserving the integrity of the initial information; and finally (3) the point of using these cells as statistical units is to allow an extremely detailed analysis while preserving total health data anonymity in the subsequent analysis.

### Weaknesses

Our approach did have certain limitations in terms of the contextual data used. Data availability necessarily constrains the variables integrated to this analysis, so that the number of contextual dimensions used to characterize neighbourhood context is also constrained.

We acknowledge that some data could not be included in our analysis. This is the case, for example, for violence in neighbourhoods, the presence of exterior annoyances and substandard housing. Traffic noise data, for instance, is considered politically sensitive when displayed at a fine scale, and we were unable to obtain access to this. The collection of data regarding quality of housing and exterior annoyances is available only for the City of Strasbourg, and is not available across the SMA scale. In addition the health data was collected between 2000 and 2008, while the contextual data was mainly available between 2007 and 2008, with the exception of the socioeconomic data, obtained from the 1999 census. The collection of data according to availability may result in a temporal gap between contextual data and its outcome data, which could influence the result observed. In our study, we are however unable to measure this misclassification.

## Conclusion

We proposed a data-driven approach developed at fine spatial scale level, aimed at the investigation of neighbourhood characteristics capable of explaining geographical distribution of the onset of MI risk. In our study, we characterized the neighbourhood free of any a priori hypothesis, and without weighting certain contextual neighbourhood components, privileging the use of diverse contextual neighbourhood profiles and the ad hoc synthetic neighbourhood areal unit. Our spatial approach allowed us to identify the neighbourhood characteristics of inhabitants living within a high MI risk cluster in comparison with those living within a low MI risk cluster.

Therefore, spatial data-driven analyses of routinely-collected data georeferenced by various sources may serve to guide policymakers in defining and promoting targeted actions at fine spatial level. Armed with local characterization of the combination between the socioeconomic dimension, social cohesion and access to amenities relating to social inequalities in health, policymakers may be able to promote more accurately-targeted actions aimed at reducing health inequalities, and promote a better understanding of social, healthy behaviour among deprived populations. An open question worthy of further research would be to determine the minimal set of data (according to the principle of parsimony and for the sake of efficiency) needed to appropriately characterize neighbourhood influences, given that what holds true in a given area may differ across geographical settings having different historical and sociological contexts.

## Appendix

See Fig. 4
